# Synthesis and Characterization of Electrical and Thermal Conductive Vinyltriethoxysilane Functionalized Graphene Oxide/Poly (Methyl Methacrylate) Nanocomposite Films

**DOI:** 10.3390/membranes13060609

**Published:** 2023-06-20

**Authors:** Srosh Fazil, Khurram Liaqat, Wajid Rehman, Magda H. Abdellatif

**Affiliations:** 1 Department of Chemistry, University of Poonch Rawalakot, Rawalakot 12350, Azad Kashmir, Pakistan; 2 Department of Chemistry, Hazara University Mansehra, Mansehra 21120, Khyber Pakhtunkhwa, Pakistan; 3 Department of Chemistry, College of Sciences, Taif University, P.O. Box 11099, Taif 21944, Saudi Arabia

**Keywords:** poly (methyl methacrylate), vinyltriethoxysilane functionalized graphene oxide, nanocomposite films

## Abstract

The present study is an attempt to improve thermal, mechanical and electrical properties of poly (methyl methacrylate) (PMMA). For this purpose, vinyltriethoxysilane (VTES) was grafted covalently on the surface of graphene oxide (GO). This VTES functionalized graphene oxide (VGO) was dispersed in the PMMA matrix using the solution casting method. The morphology of the resultant PMMA/VGO nanocomposites was analyzed by SEM indicating well-dispersed VGO in the PMMA matrix. Thermal stability, tensile strength and thermal conductivity increased by 90%, 91% and 75%, respectively, whereas volume electrical resistivity and surface electrical resistivity reduced to 9.45 × 10^5^ Ω/cm and 5.45 × 10^7^ Ω/cm^2^, respectively.

## 1. Introduction

Poly (methyl methacrylate) (PMMA) is a thermoplastic polymer that is used in a variety of different areas due to its low cost and unique properties [1,2]. Its amorphous form, biocompatibility, optical clarity and low susceptibility to ultraviolet light has prompted great interest in it [3]. Due to its environmental stability, PMMA is preferred over polyethylene and polystyrene for various outdoor applications in the plastics industry [1,2,3,4,5]. The inclusion of nano-fillers can improve the electrical, thermal and mechanical characteristics of PMMA since it is thought to be an excellent host for them. Among the different classes of nano-fillers, graphene oxide is preferred because of the presence of various oxygen-containing functional groups on its surface which can be functionalized with various materials for tailoring the interface between polymer and filler [4,5,6,7,8]. Surface modification of nanomaterials with organic moiety also aids in the prevention of agglomeration by enhancing the nanomaterial dispersion in polymer matrices [9]. Silane is a helpful coupling agent for nanomaterial modification. Different research groups reported various forms of silane as reinforcements in PMMA-based composites.

M-E Vlachopoulou and his co-workers have used 3-aminopropyltriethoxysilane for the formation of a silicon-containing bond on the surface of PMMA through O_2_ plasma activation of the surfaces, which has resulted in irreversible bonding [10].

Andri K. Riau and his co-workers have modified the surface of PMMA with a calcium phosphate coating (p-CaP), dopamine, followed by CaP (d-CaP) with 3-aminopropyltriethoxysilane (3-APTES), and plasma to solve the problem of poor adhesion between PMMA and soft corneal tissue. Surface modifications produced significantly improved interfacial adhesion strength compared to untreated PMMA. Longer-term stability of the adhesion was achieved by d-CaP [11].

Siu-Ming Yuen synthesized a PMMA–VTES copolymer by mixing methyl methacrylate monomer and VTES in the presence of an initiator. This copolymer solution was mixed with 3-isocyanato–propyltriethoxysilane (IPTES)-functionalized MWCNTs in the presence of dibutyltindilaurate as the catalyst. The resulting nanocomposites showed enhanced thermal stability and thermal conductivity [12].

We have created covalent bonding between graphene oxide (GO) and vinyltriethoxysilane (VTES), this vinyltriethoxysilane-functionalized graphene oxide (VGO) was used for the formation of nanocomposites with PMMA through the solution casting method. To the best of our knowledge, these nanocomposites of PMMA have never been reported previously. These nanocomposites were characterized to evaluate the effects of VGO on reinforcement of the properties of PMMA.

## 2. Experimental Section

### 2.1. Materials and Methods

Vinyltriethoxysilane (VTES), N, N-dimethylformamide (DMF), triethylamine (TEA), methanol and poly (methyl methacrylate) (PMMA) were purchased from Sigma-Aldrich USA.

### 2.2. Synthesis of VGO

**Scheme 1:**
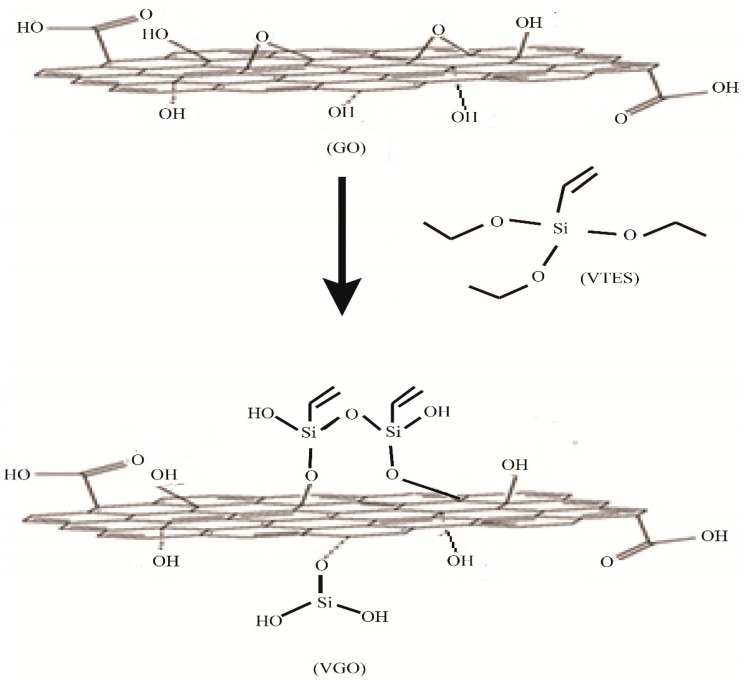
Synthesis of VGO.

In total, 1.20 g VTES and 0.30 mL of triethylamine (TEA) were added in a dispersion of GO in DMF and agitated for 30 min. It was then refluxed under nitrogen at 70 °C for 24 h. Next, 100 mL methanol was added to this mixture after 24 h to dilute the residue and was then subjected to filtration and washing with water and methanol to obtain VGO. Thus was then dried in the vacuum oven [13] [Scheme 1].

### 2.3. Synthesis of GO and VGO/PMMA Nanocomposite Films

**Scheme 2:**
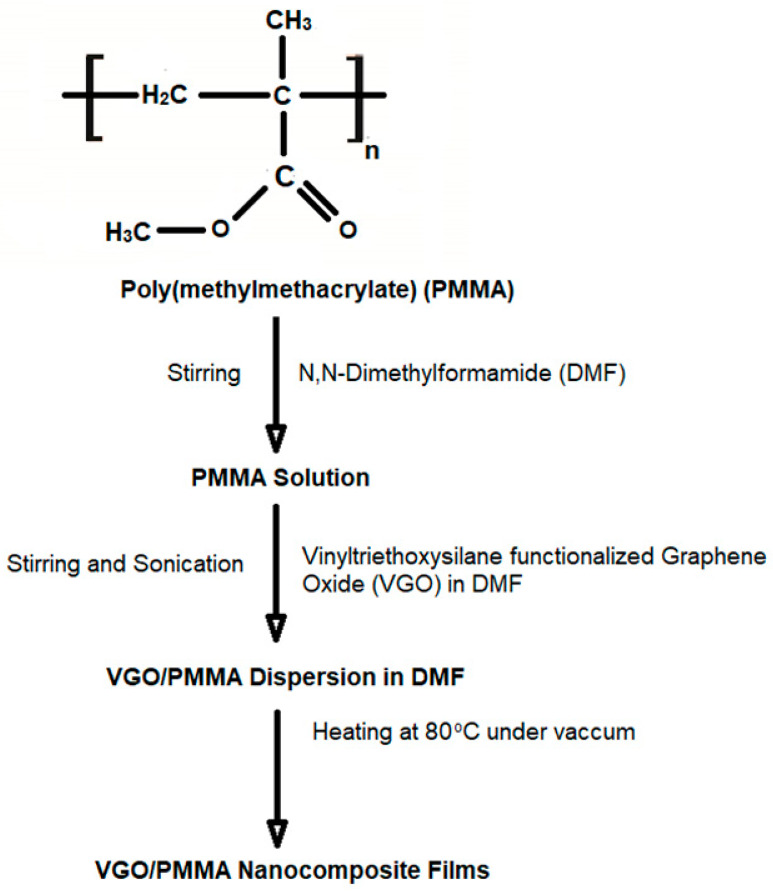
Synthesis of VGO/PMMA nanocomposite films.

For nanocomposite fabrication, solution mixing was utilized. PMMA was mixed with VGO or GO that had been disseminated in DMF ultrasonically and sonicated for 4 h. This dispersion was transferred into a Petri dish and subjected to vacuum drying at 80 °C for 12 h to obtain VGO/PMMA nanocomposite films. PMMA nanocomposite films with 0.2, 0.4, 0.8, 1.0, 2.0 and 3 wt% VGO were synthesized [Scheme 2].

### 2.4. Characterization

X-ray photoelectron spectroscopic analysis was carried out by Kratos Axis Ultra DLD spectrometer. Smart lab x-ray diffractometer was used for x-ray diffraction studies. FTIR spectra of nanocomposite films were obtained by using a Nicolet Avatar 320 FTIR spectrometer in the range of 4000 and 400 cm^−1^. TA 2000 was used for measurement of thermal stability of PMMA/VGO nanocomposite. Hitachi scanning electron microscope FESEM (S-4200) was used to evaluate morphological features of nanocomposites. An ULTRA Mesohmeter SM-8220 (from DKK TOA Corporation, Tokyo, Japan) was used to evaluate surface and volume electrical resistance. After adding varying amounts of VTES, the electrical resistivity of surface and volume were evaluated. For measuring thermal conductivity, H940 heat conduction unit was used.

## 3. Results and Discussion

### 3.1. Characterization of VGO

**Figure 1:**
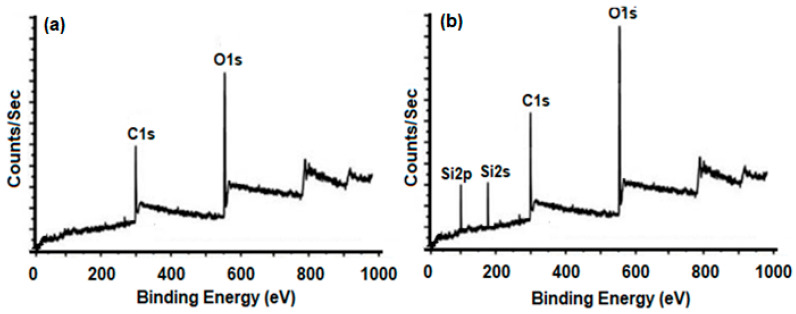
XPS survey scan of (**a**) GO and (**b**) VGO.

We have used x-ray photoelectron spectroscopy (XPS) for confirming the functionalization of GO. For this purpose, we have correlated XPS spectra of VGO with GO. When correlated with the survey scan of GO, the XPS survey scan of VGO indicates the presence of two new peaks, ascribed as Si2s and Si2p (Figure 1). This verifies the presence of VTES on the surface of GO [14].

**Figure 2:**
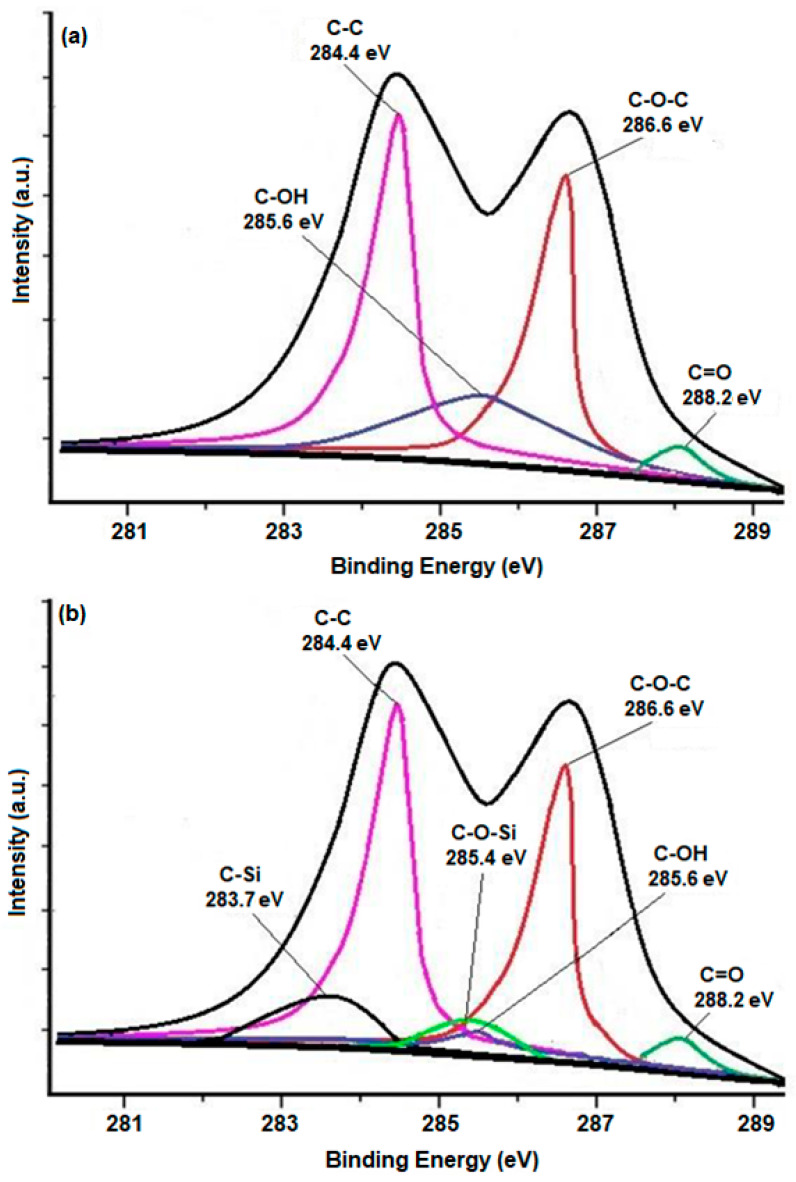
C1s spectrum of (**a**) GO and (**b**) VGO.

In order to evaluate the attachment mechanism of VTES onto GO, XPS C1s spectrum evaluation was utilized. The emergence of two additional peaks at 285.4 eV and 283.7 eV corresponding to C-O-Si and C-Si, and a reduction in the intensity of peak corresponding to the hydroxyl group in the C1s spectrum of VGO (Figure 2b) when correlated with the C1s spectrum of GO (Figure 2a), indicates the interaction between VTES and hydroxyl groups of GO [15,16].

**Figure 3:**
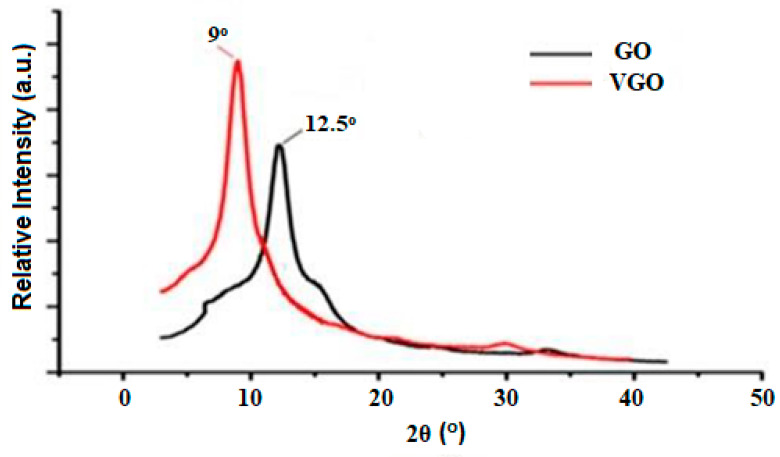
XRD pattern of VGO and GO.

X-ray diffraction (XRD) was used for further validating the VTES functionalization of graphene oxide. According to Figure 3, the diffraction angle (2°) decreases from 12.5° for GO to 9° for VGO, increasing interlayer spacing. The presence of VTES groups on the surface of GO accounts for these changes in diffraction angle (2θ) and interlayer spacing. Successful functionalization of GO with VTES is evidenced by the disappearance of a peak in the VGO diffraction pattern at 2 = 12.5° [17,18].

**Figure 4:**
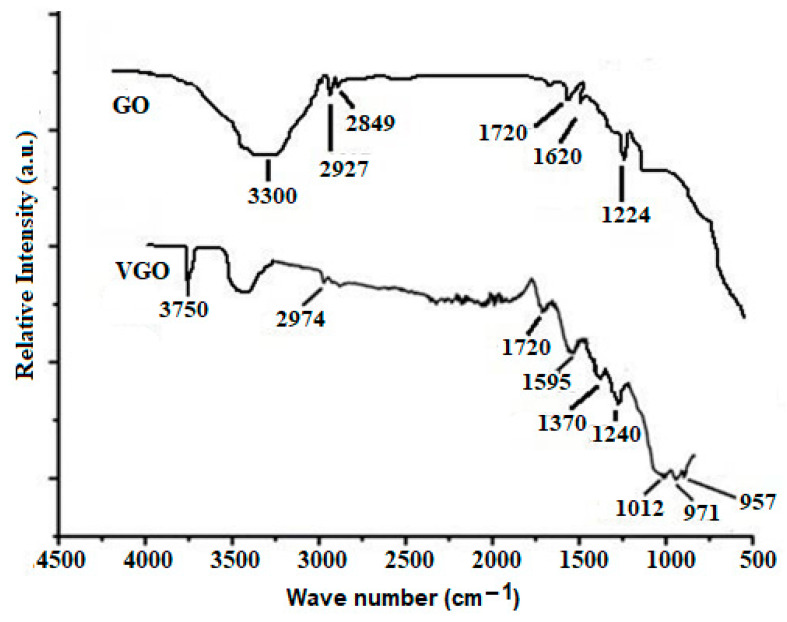
ATR-FTIR spectra of VGO and GO.

Figure 4 shows the attenuated total reflection Fourier transform infrared (ATR-FTIR) spectra of GO and VGO. It appears in GO spectrum peaks at 1224 (C-OH stretch of alcohol group), 1620 (C=C stretch of unoxidized graphitic domain), 1720 (C=O stretch of carboxyl group), 2849 and 2927 cm^−1^ (due the asymmetric and symmetric CH_2_ stretching of GO). However, in the spectrum of VGO peaks, it appears at 957 cm^−1^, 2974 cm^−1^ and 1595 cm^−1^ due to the -CH_2_ group of Si–CH=CH_2,_ CH stretching vibration of vinyl and C=C groups of VTES, respectively. Stretching of Si-O-C and Si–O–Si gives rise to peaks at 971 cm^−1^ and 1012 cm^−1^, respectively.

The band at 3300 cm^−1^ is due to OH groups on the surface of GO that have not been used in bond formation between GO and VTES. The peak at 3750 cm^−1^ corresponds to the presence of Si-OH at the terminal end of the Si-O-Si network. These peaks are consistent with earlier research [19,20,21].

### 3.2. Characterization of PMMA/VGO Nanocomposite Films

**Figure 5:**
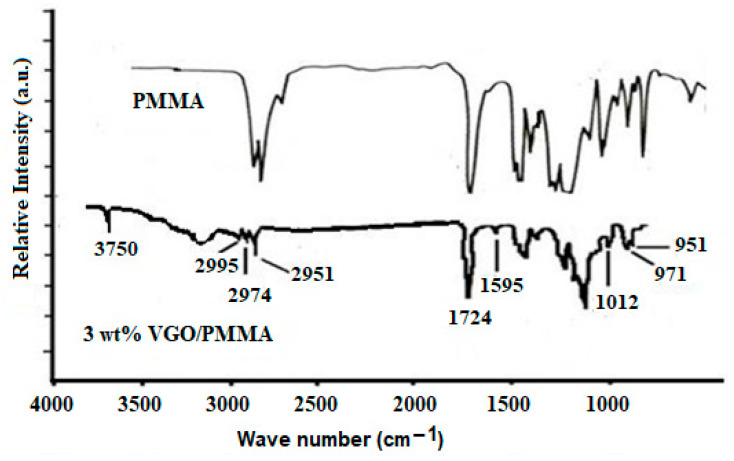
ATR-FTIR spectrum of PMMA and 3 wt% VGO/PMMA nanocomposite film.

By comparing the attenuated total reflection Fourier transform infrared (ATR-FTIR) spectra of PMMA and 3 wt% PMMA/VGO nanocomposite film (Figure 5), it appears that the PMMA/VGO nanocomposite film spectra have peaks of PMMA (2995 and 2951 cm^−1^ for C–H stretching, 1724 cm^−1^ for C=O stretching, 1200 and 1148 cm^−1^ for C–O stretching) and VGO as discussed earlier. There is also band at 3200 cm^−1^ corresponding to OH stretching which has shifted to lower frequency, indicating hydrogen bonding between VGO and PMMA [22].

**Figure 6:**
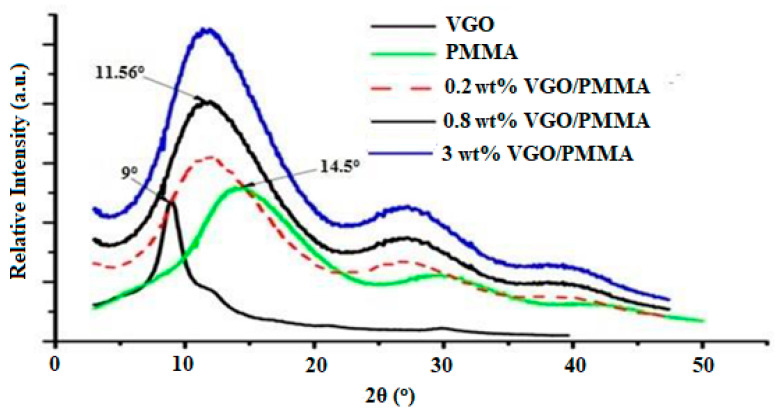
XRD patterns of VGO, PMMA and VGO/PMMA nanocomposite films with varying VGO contents.

In the x-ray diffraction (XRD) patterns (Figure 6), peaks at 2θ = 9°, 14.5° and 11.56° correspond to VGO, PMMA and VGO/PMMA nanocomposite films, respectively. The peak intensity owing to the nanocomposite increases as the VGO content increases, indicating that VGO is present in the nanocomposite. Diffraction patterns of VGO/PMMA nanocomposites do not show any peak that corresponds to VGO (9°) or PMMA (14.5°), suggesting exfoliation, as previously described [17,18].

**Figure 7:**
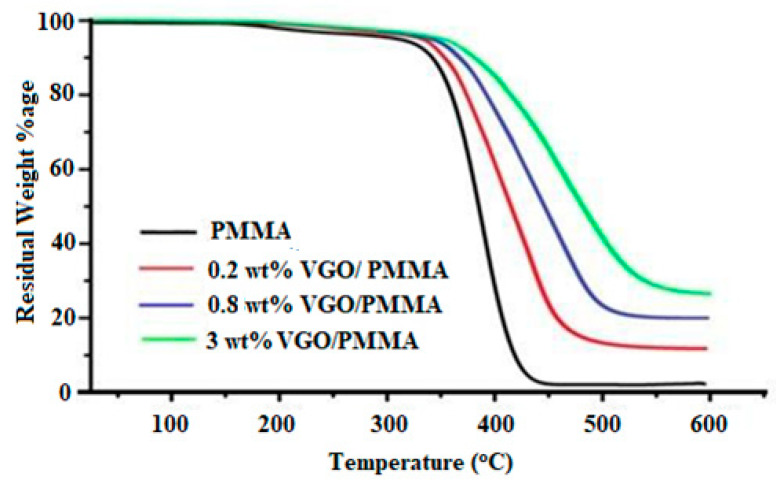
TGA curves of PMMA and VGO/PMMA nanocomposite films.

Thermogravimetric analysis (TGA) was performed under nitrogen at temperatures ranging from 25 to 600 °C. Figure 7 shows that VGO/PMMA nanocomposite films have better thermal stability than PMMA and that as the VGO component of the nanocomposite rises, so does the thermal stability of the nanocomposite. The thermal stability of the nanocomposite is usually calculated at a temperature that results in a 5.0% weight loss. In this typical scenario for a 5% weight loss, the temperature rises by 11.20 °C, 23.70 °C and 33.95 °C for 0.20, 0.80 and 3.0 wt% VGO/PMMA nanocomposite films, respectively, as compared to PMMA. Figure 7 indicates that weight loss decreases from 88.50% to 73.70% as VGO loading increases from 0.20 to 3.0 wt percent, demonstrating a reduction in the rate of breakdown due to the inclusion of VGO into nanocomposites. In general, higher Si concentration and higher Si-O-Si (due to VGO) content are linked to increased thermal stability. The Si-O-Si functional group may prevent the thermal disintegration of the polymer matrix [12].

**Figure 8:**
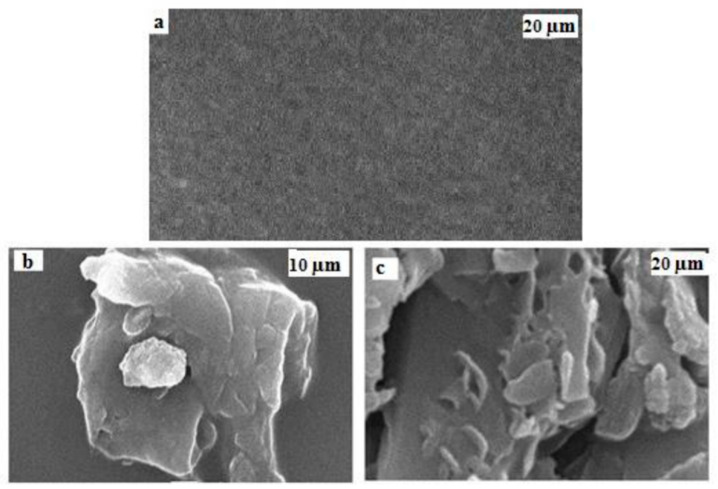
SEM images of (**a**) PMMA (**b**,**c**) 3 wt% VGO/PMMA nanocomposite film.

Scanning electron microscopic analysis reveals that the surface of neat PMMA is smooth (Figure 8a), whereas, in the case of 3.0 wt% VGO/PMMA film (Figure 8b,c), agglomerations can be detected. These agglomerations are caused by the self-linking of silanols group as VGO content increases.

Effects of various VGO loadings on the mechanical characteristics of nanocomposite films are presented in Table 1. Tensile strength and Young’s modulus increase up to 0.8 wt% of VGO by 91.95% and 94.98%, and after that it decreases, whereas elongation at break decreases as compared to PMMA. The existence of lumps, as shown in images of SEM in Figure 8b,c, might explain this drop in mechanical property values at high VGO concentration. Based on these findings, it appears that the 0.8 wt% VGO/PMMA nanocomposite film is mechanically better than all other VGO/PMMA nanocomposite films.

**Figure 9:**
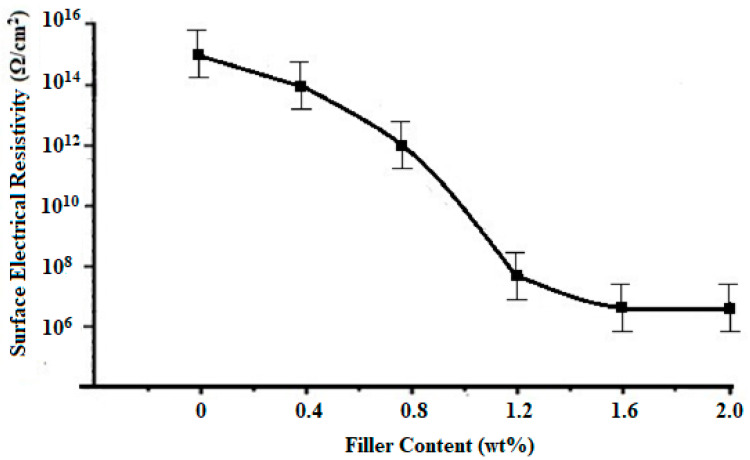
Surface electrical resistivity of VGO/PMMA nanocomposite films with varying VGO contents.

**Figure 10:**
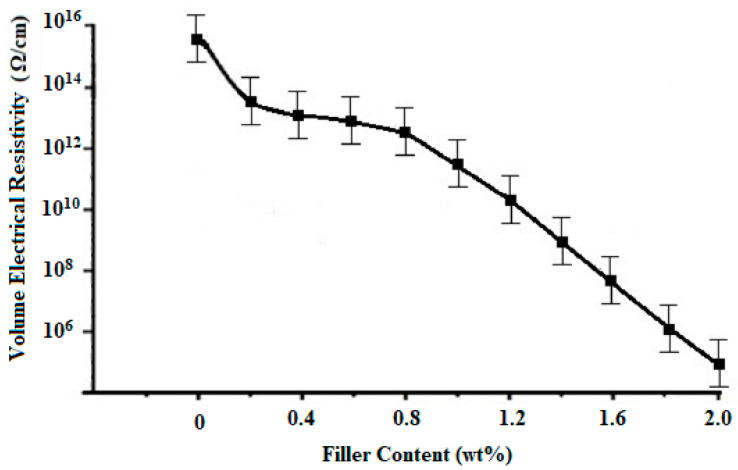
Volume electrical resistivity of VGO/PMMA nanocomposite films with varying VGO contents.

**Figure 11:**
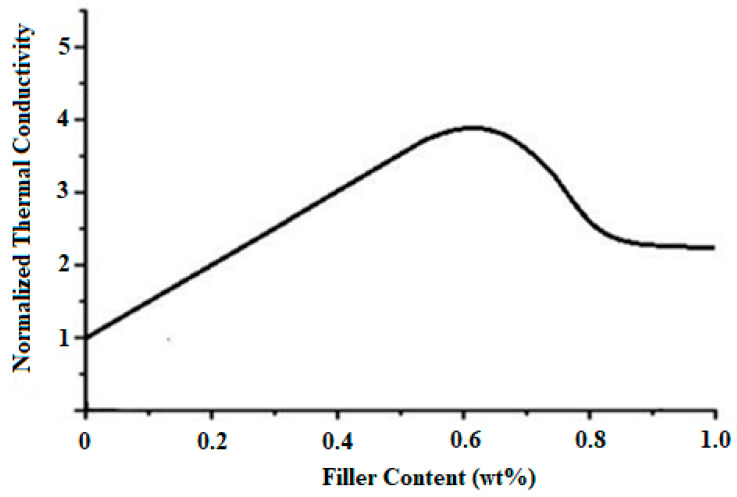
Normalized thermal conductivity of VGO/PMMA nanocomposite films with varying VGO contents.

According to Figure 9, the surface electrical resistivity of neat PMMA is 1.00 × 10^15^ Ω/cm^2^, with the increase in the contents of VGO from 0–1.2 wt%, surface electrical resistivity of VGO/PMMA nanocomposite films decreases to 5.45 × 10^7^ Ω/cm^2^, after which there is no significant change. Figure 10 shows the change in volume electrical resistivity with the change in content of VGO. The volume electrical resistivity of neat PMMA is 1.00 × 10^16^ Ω/cm. With the increase in the VGO contents from 0.2–2 wt%, volume electrical resistivity decreases to 9.45 × 10^5^ Ω/cm. Plotted against VGO concentration in Figure 11 is the normalized thermal conductivity of PMMA/VGO nanocomposite films. The normalized thermal conductivity of plain PMMA is 1 [23]. It increases when the VGO concentration rises to 0.6 wt% after which it decreases slightly. This drop might be attributed to a decrease in the degree of condensation of the T distribution, which corresponds to ease in polymer mobility. Energy that travels through the polymer may be consumed, resulting in the decrease in thermal conductivity of nanocomposite films [12].

## 4. Conclusions

A covalent bond between VTES and the hydroxyl group of GO was confirmed by XPS. The incorporation of VGO into PMMA has resulted in a chemical interaction between VGO and PMMA. SEM microphotographs show the uniform dispersibility of VGO into the PMMA matrix enabling reinforcement in the properties of the resulting VGO/PMMA nanocomposites. Mechanically, the PMMA/VGO nanocomposite films were superior to PMMA. Surface electrical resistivity and volume electrical resistivity decreased as the VGO content increased. Thermal conductivity increased with the VGO content and maximal at 0.6 wt% VGO content.

## Figures and Tables

**Table 1:** Tensile Data.

Sample	Tensile Strength (MPa)	Young’s Modulus (MPa)	Elongation at Break
PMMA	46.5 ± 0.01	5.11 ± 0.02	9.1 ± 0.01
0.2% VGO/PMMA Nanocomposite film	200 ± 0.03	31.74 ± 0.02	6.3 ± 0.03
0.8% VGO/PMMA Nanocomposite film	550 ± 0.02	101.85 ± 0.01	5.4 ± 0.01
3% VGO/PMMA Nanocomposite film	435 ± 0.01	87.00 ± 0.03	5.0 ± 0.02

## Data Availability

The data presented in this study are available on request from the corresponding author.
